# Influence of *Miscanthus floridulus* on Heavy Metal Distribution and Phytoremediation in Coal Gangue Dump Soils: Implications for Ecological Risk Mitigation

**DOI:** 10.3390/plants14060836

**Published:** 2025-03-07

**Authors:** Jiaolong Wang, Yan Jiang, Yuanying Peng, Xiaoyong Chen, Wende Yan, Xiaocui Liang, Qian Wu, Jingjie Fang

**Affiliations:** 1 School of Materials and Chemistry Engineering, Pingxiang University, Pingxiang 337055, China; pxuwjl@126.com (J.W.); jiangyan890317@gmail.com (Y.J.); 2 College of Arts and Sciences, Lewis University, Romeoville, IL 60446, USA; pengyu@lewisu.edu; 3 College of Arts and Sciences, Governors State University, University Park, IL 60484, USA; 4 College of Life and Environmental Science, Central South University of Forestry and Technology, Changsha 410004, China; t20081388@csuft.edu.cn (X.L.); fjj517@163.com (J.F.); 5 National Engineering Laboratory of Applied Technology for Forestry & Ecology in Southern China, Central South University of Forestry and Technology, Changsha 410004, China; 6 Key Laboratory of Subtropical Forest Ecology of Hunan Province, Changsha 410004, China; 7 Faculty of Resources and Environmental Engineering, Anshun University, Anshun 561000, China; csfuwq@163.com

**Keywords:** coal gangue disposal, heavy metal pollution, rhizosphere remediation, *Miscanthus floridulus*, root exudates

## Abstract

Coal gangue dumps, a byproduct of coal mining, contribute significantly to heavy metal contamination, impacting soil and water quality. In order to assess the levels of heavy metal contamination in soils at different stages of abandonment, this study investigated the role of *Miscanthus floridulus* (*M. floridulus*) in the spatial distribution and remediation of six heavy metals (Cd, Cr, Mn, Ni, Cu, and Pb) in coal gangue dump soils abandoned for 0, 8, and 12 years in Pingxiang City, Jiangxi Province, China. Fieldwork was conducted at three sites operated by the Pingxiang Mining Group: Anyuan (active, barren), Gaokeng (8 years, natural vegetation), and Qingshan (12 years, partially remediated). Anyuan remains largely barren, while Gaokeng supports natural vegetation without formal remediation. In contrast, Qingshan supports diverse plant species, including *M. floridulus*, due to partial remediation. Using a randomized design, root exudates, heavy metal concentrations, and soil properties were analyzed. The results showed that Cd poses the highest ecological risk, with concentrations of 64.56 mg kg^−1^ at the active site, 25.57 mg kg^−1^ at the 8-year site, and 39.13 mg kg^−1^ at the 12-year site. Cu and Pb showed accumulation, while Cr and Mn decreased over time. Root exudates from *M. floridulus* enhanced metal bioavailability, influencing Cd, Cr, and Ni concentrations. These findings highlight the importance of rhizosphere processes in metal mobility and inform sustainable remediation strategies for post-mining landscapes.

## 1. Introduction

Coal mining, a critical component of global energy production, generates significant byproducts, including coal gangue, which accumulates in dumps and poses severe environmental risks. These dumps are often contaminated with heavy metals like Cd, Cr, Mn, Ni, Cu, and Pb, which can leach into soils and water systems, leading to ecological degradation and public health concerns [1,2,3]. The extensive accumulation of over 7 billion tons of coal gangue in China alone highlights the scale of this issue, with approximately 2600 gangue hills covering 1.5 million hectares of land [4,5]. In addition to direct contamination, spontaneous combustion of gangue piles releases harmful gases, exacerbating air pollution and respiratory health problems [6,7].

Phytoremediation is an environmentally friendly and cost-effective technology that utilizes plants to remove, degrade, or stabilize contaminants in soil and water, making it an attractive solution for mitigating environmental pollution in post-mining landscapes. Specifically, plants can assist in the reduction of heavy metal contamination through various mechanisms such as phytoextraction, phytostabilization, and rhizodegradation. Heavy metals, including cadmium (Cd), lead (Pb), nickel (Ni), copper (Cu), and chromium (Cr), are widespread contaminants in coal mining areas, posing significant risks to both environmental and human health. These metals can persist in soil for extended periods, posing a threat to soil fertility, microbial diversity, and plant health. The mobility of heavy metals is influenced by factors such as pH, soil texture, organic matter content, and the presence of plant roots and their exudates [8,9]. Root exudates from plants like *Miscanthus floridulus* can alter the bioavailability and mobility of heavy metals by releasing organic acids, chelating agents, and other compounds that either facilitate or inhibit metal uptake by plants. These processes make phytoremediation an important tool for reducing ecological risks in contaminated areas, promoting long-term restoration of ecosystem functions [10].

The contamination of soils by heavy metals diminishes soil fertility and disrupts ecosystem stability, complicating restoration efforts in mining-impacted areas [4,5]. While traditional methods such as resource recycling and safe disposal have been explored, they often fall short of providing sustainable solutions [7,11]. In response, phytoremediation has emerged as a promising and sustainable technique for mitigating heavy metal contamination in soils. This approach leverages plants to stabilize, accumulate, or transform contaminants, reducing their bioavailability and ecological impact [8,12,13]. Key factors, such as rhizosphere interactions, plant growth-promoting rhizobacteria (PGPR), and root exudates, enhance phytoremediation by increasing metal mobility, shaping microbial communities, and promoting soil recovery [14,15,16]. Despite the progress made in phytoremediation research, significant gaps remain. For example, the specific role of root exudates in mitigating heavy metal contamination in coal gangue soils has not been fully explored. Root exudates, which can influence the mobility of metals and microbial activity, have been studied in general soil contexts, but not under the stress of coal mining waste. Furthermore, while previous studies have investigated the concentrations of heavy metals in soils, few have compared these concentrations in rhizosphere or non-rhizosphere soils of plants in coal mining environments, limiting our understanding of how plant roots directly influence soil remediation.

Among potential phytoremediation species, *Miscanthus floridulus* has shown significant promise due to its robust growth, extensive root system, and ability to adapt to harsh mining waste environments. Its rhizosphere processes, particularly the production of root exudates, play a crucial role in influencing the mobility and bioavailability of heavy metals, aiding in their stabilization or uptake [6,17,18]. Recent research has underscored the synergistic role of PGPR and plant exudates in modulating rhizosphere microbial communities, thereby enhancing the remediation efficiency of hyperaccumulator plants [13,19,20].

This study sought to address gaps in understanding the role of plant roots, particularly *M. floridulus*, in mitigating heavy metal contamination in coal gangue-contaminated soils. By analyzing the impact of root exudates on metal mobility and bioavailability, comparing heavy metal concentrations between the rhizosphere and non-rhizosphere soils, and evaluating soil pollution risks using ecological indices, the research aimed to contribute valuable insights into phytoremediation processes. We hypothesized that *Miscanthus floridulus* plays a significant role in mitigating heavy metal contamination in coal gangue-contaminated soils by influencing metal mobility and bioavailability through its root exudates. Specifically, we expected that root exudates would enhance the bioavailability of metals in the rhizosphere, leading to higher concentrations of heavy metals such as Cd, Cr, Mn, Ni, Cu, and Pb in the rhizosphere soils compared to non-rhizosphere soils. Additionally, we anticipated that the presence of *M. floridulus* would contribute to a reduction in ecological risk by altering the distribution and bioavailability of these metals, thus supporting the potential for phytoremediation in coal gangue dump sites. The study’s objectives were: (1) to analyze the concentrations of six heavy metals—Mn, Cd, Cr, Ni, Cu, and Pb—in both rhizosphere and non-rhizosphere soils; (2) to examine the composition of root exudates under heavy metal stress and their influence on soil properties; (3) to assess differences in heavy metal concentrations between rhizosphere and non-rhizosphere soils of dominant plants; and (4) to evaluate soil pollution risks using indices such as the single-factor pollution index, the Nemero comprehensive pollution index, and the potential ecological hazard index. By addressing these gaps, the study aimed to advance strategies for ecological restoration in coal mining areas and promote sustainable, plant-based solutions for restoring contaminated landscapes.

## 2. Results

### 2.1. Spatial Distribution of Heavy Metals (Cd, Pb, Zn, Cu, Cr, Ni) in Soils at Varying Distances from M. floridulus Roots Across Three Coal Gangue Dump Sites

**Figure 1:**
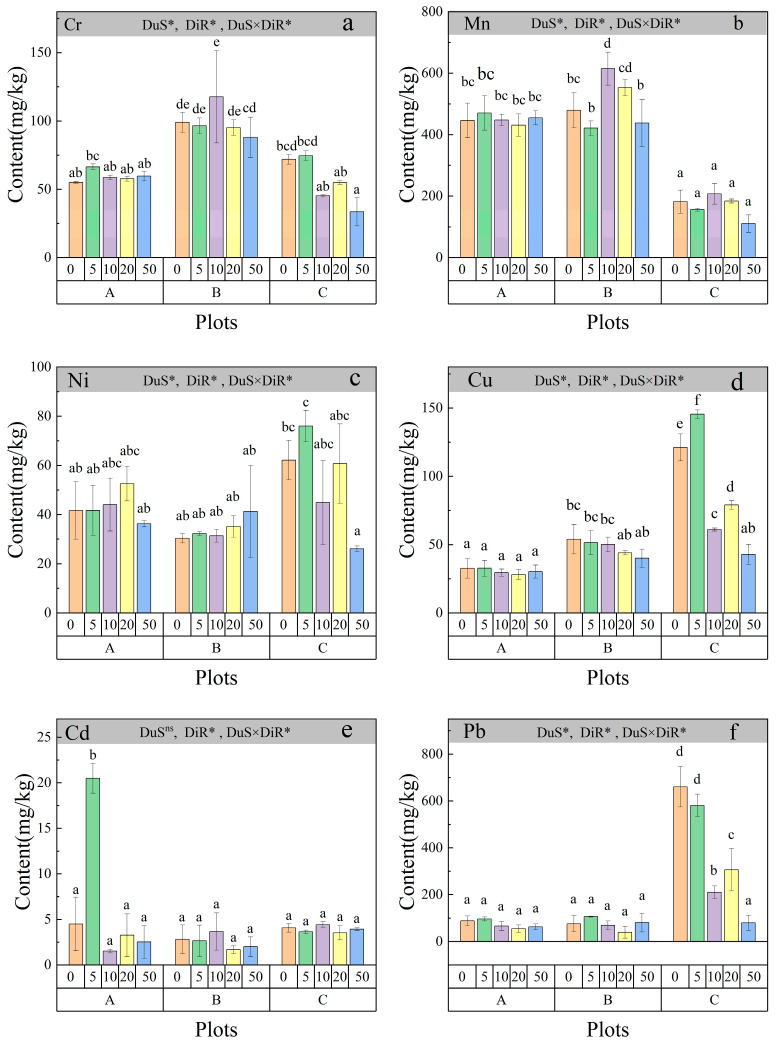
Distribution of soil heavy metal content in coal gangue dump sites with varying years of abandonment. A, B, and C represent the Anyuan Coal Mine, Gaokeng Coal Mine, and Qingshan Coal Mine coal gangue dumps in Pingxiang City, respectively. At each site, the dominant plant, *M. floridulus*, was selected for root exudate measurements. Soil samples were collected from both the rhizosphere (0 cm from the roots) and non-rhizosphere at distances of 5 cm, 10 cm, 20 cm, and 50 cm from the roots to determine heavy metal content. Different uppercase letters indicate significant differences among dump sites, and different lowercase letters indicate significant differences among distances from both the rhizosphere and non-rhizosphere (*p* < 0.05, Tukey’s test). * Significant at *p* < 0.05 level, ns—not significant. Columns represent the mean ± standard error (n = 3). Abbreviations: DuS, dump site; DiR, distance from both the rhizosphere and non-rhizosphere. Specifically, (**a**), (**b**), (**c**), (**d**), (**e**), and (**f**) represent the heavy metals Cr, Mn, Ni, Cu, Cd, and Pb, respectively.

Figure 1 shows the distribution of heavy metals (Cd, Pb, Zn, Cu, Cr, and Ni) in soils from three coal gangue dump sites with varying closure durations: Anyuan Coal Mine (0 years, operational), Gaokeng Coal Mine (8 years), and Qingshan Coal Mine (12 years). Measurements were taken in rhizosphere soils (0 cm) and at 5, 10, 20, and 50 cm from the roots. The distribution of soil heavy metal content showed significant differences (*p* < 0.05) among the coal gangue dump sites (DuS), the distances from both the rhizosphere and non-rhizosphere (DiR), and their interaction (DuS × DiR) (Figure 1). Heavy metal content, including Cr (33.47–129.18 mg kg^−1^) (Figure 1a), Mn (110.29–614.59 mg kg^−1^) (Figure 1b), Ni (26.03–75.98 mg kg^−1^) (Figure 1c), Cu (28.10–145.46 mg kg^−1^) (Figure 1d), Cd (1.51–20.05 mg kg^−1^) (Figure 1e), and Pb (39.32–660.34 mg kg^−1^) (Figure 1f), varied significantly (*p* < 0.05) across sites and distances. Cr content initially increased before decreasing with prolonged closure times. At Anyuan (Plot A), Cr levels remained relatively stable, ranging from 50 to 75 mg kg^−1^. In contrast, Gaokeng (Plot B) exhibited a peak concentration of approximately 140 mg kg^−1^, indicative of legacy contamination. At Qingshan (Plot C), Cr content decreased to around 40 mg kg^−1^ (Figure 1a). Mn content was the highest and showed the greatest variability among the plots. At Plot A, Mn levels remained stable between 400 and 500 mg kg^−1^. In Plot B, content peaked at approximately 600 mg kg^−1^, while in Plot C, Mn levels declined over time (Figure 1b). Ni content varied by site: Plot A remained stable at 30–50 mg kg^−1^, Plot B peaked at around 70 mg kg^−1^, and Plot C exhibited an initial peak followed by a decline to approximately 40 mg kg^−1^ (Figure 1c). Cu and Pb content increased with longer closure durations, with Plot C showing the highest content (Figure 1d,f). Cd content decreased initially and then increased over time (Figure 1e). Metal content was highest near the roots (5–10 cm) and decreased with distance, with Mn peaking at 10 cm and Pb steadily decreasing as distance increased.

### 2.2. Assessment of Soil Heavy Metal Contamination and Potential Ecological Risks Across the Three Study Sites

The single-factor pollution index values for the six analyzed metals (Cr, Mn, Ni, Cu, Cd, and Pb) varied significantly across the three coal gangue dumps (A: Anyuan Coal Mine, B: Gaokeng Coal Mine, C: Qingshan Coal Mine in Table 1 (*p* < 0.05)). Cr contamination was highest at site B (5.17), significantly exceeding levels at sites A (1.238) and C (1.166). Mn pollution was comparable at sites A (1.736) and B (1.934), but significantly lower at site C (0.648) (*p* < 0.05). Ni showed the highest index at site C (2.84) (*p* < 0.05), followed by site A (2.276) and site B (1.794) (*p* < 0.05). Cu contamination was greatest at site C (4.32) (*p* < 0.05), differing significantly from sites A (1.472) and B (2.302) (*p* < 0.05), which were similar. Cd pollution was the most severe at site A (64.56), followed by site C (39.134) and site B (25.574) (*p* < 0.05). Pb levels were highest at site C (11.444), while sites A (2.286) and B (2.306) exhibited comparable lower contamination (*p* < 0.05).

The Nemero comprehensive pollution index values for the six metals (Cr, Mn, Ni, Cu, Cd, and Pb) varied significantly among the three coal gangue dumps (A: Anyuan, B: Gaokeng, C: Qingshan) (*p* < 0.05, Table 2). Cr pollution was highest at site B (2.27) compared to sites A (1.41) and C (1.37), which showed no significant difference (*p* > 0.05). Mn levels were similar at sites A (1.78) and B (2.16), but significantly lower at site C (0.73) (*p* < 0.05). Ni pollution was highest at site C (3.47), followed by site A (2.54), with site B (1.99) significantly lower (*p* < 0.05). Cu levels were highest at site C (5.81), differing significantly from sites A (1.52) and B (2.45), which were comparable (*p* < 0.05). Cd pollution was most severe at site A (152.00), followed by site C (41.74) and site B (31.57), all significantly different (*p* < 0.05). Pb contamination was highest at site C (16.64), while sites A (2.81) and B (2.85) showed similar lower levels (*p* < 0.05).

The results from the potential ecological hazard index evaluation (Table 3 and Table 4) revealed significant variations in heavy metal contamination across the three coal gangue dumps (A: Anyuan, B: Gaokeng, C: Qingshan). Cr contamination was highest at site B (4.14), followed by site A (2.48) and site C (2.33), with site B showing significantly higher levels. Ni levels were highest at site C (14.19), followed by site A (11.38), and site B had the lowest levels (8.98). Cu contamination was highest at site C (21.61), significantly differing from both sites A (7.36) and B (11.52), which did not differ from each other. Cd contamination posed the highest ecological risk among all metals, with site A (1936.9) showing the highest individual risk index (Eri) for Cd, followed by site C (1174) and site B (767.22). Pb levels were highest at site C (57.2), significantly higher than at sites A (11.43) and B (11.53b), which exhibited similar levels. The cumulative ecological risk index (RI) further highlighted Cd’s dominance in contributing to ecological hazards, with site A exhibiting the highest RI (9684.5), followed by site C (5870) and site B (3836.1).

While Cd posed the most significant ecological threat, Cr, Mn, Ni, Cu, and Pb posed moderate to low ecological risks. However, at site C, elevated levels of Ni (14.19) and Cu (21.61) indicated their potential contribution to overall ecological risk, though not as critical as Cd. Pb also showed a notable increase at site C, with an Eri of 57.2. These findings demonstrate the varying trends in heavy metal pollution based on mine closure duration, with Cu and Pb showing continuous accumulation, while Cr and Mn followed a rise-and-fall pattern. Cd and Ni showed an initial decline in risk levels before increasing again.

## 3. Discussion

### 3.1. Spatial Distribution of Heavy Metals (Cd, Pb, Zn, Cu, Cr, and Ni) in Soils Across the Three Coal Gangue Dump Sites at Different Distances from the Roots of M. floridulus

The observed variations in heavy metal content across the three coal gangue dump sites (Anyuan, Gaokeng, and Qingshan coal mines) reflect the combined effects of site history, closure duration, and plant-mediated transformations in soil chemistry. Chromium (Cr) content initially increased and then declined over time, suggesting potential leaching or immobilization processes. At the newly operational site (Plot A), Cr remained stable due to continuous coal gangue deposition. The peak at Plot B (8 years) likely reflects legacy contamination from past mining activities, whereas the decline at Plot C (12 years) suggests Cr stabilization through plant uptake and immobilization in organic matter [21]. Additionally, Cr reduction over time may result from adsorption onto iron oxides and organic matter, a process observed in reclaimed mine soils [22]. Similarly, manganese (Mn) exhibited the highest content and fluctuations among the metals studied, with a peak at 600 mg kg^−1^ in Plot B, potentially due to redox transformations influencing Mn bioavailability [23]. The decline at Plot C suggests Mn uptake by *M. floridulus*, a known Mn accumulator in phytoremediation settings [24]. Since Mn solubility is pH-dependent, alterations in soil pH due to root exudates may further regulate its availability over time. Nickel (Ni) content showed site-specific trends, with peaks at Plot B likely reflecting residual contamination from coal waste, while the decline at Plot C suggests sequestration in organic-rich fractions or uptake by vegetation [25]. Given Ni’s association with iron and manganese oxides, its relative stability at Plot A may be due to ongoing gangue deposition without significant remediation [26].

In contrast to other metals, copper (Cu) and lead (Pb) content increased with prolonged closure durations, particularly in Plot C, indicating their persistence and strong affinity for organic matter and clay minerals [27]. The gradual enrichment of Pb suggests reduced leaching and enhanced sorption onto soil colloids or incorporation into stable organic–mineral complexes [28]. The role of *M. floridulus* in enhancing Pb and Cu stabilization via rhizosphere processes warrants further investigation. Cadmium (Cd) dynamics exhibited a distinct pattern, initially decreasing before increasing over time, reflecting a complex interplay of leaching, plant uptake, and redistribution in the soil matrix. In early-stage sites (Plot A), Cd mobility may be enhanced due to weak adsorption properties, leading to an initial decline. However, as organic matter accumulates and root exudates alter metal bioavailability, Cd retention in surface soils may increase [29]. The enhanced mobility of Cd under acidic conditions could further contribute to its redistribution, emphasizing the need for long-term monitoring to assess its ecological risk.

Heavy metal content was highest near plant roots (5–10 cm) and decreased with distance, highlighting the strong influence of rhizosphere processes such as root exudation, microbial activity, and metal uptake by *M. floridulus* [30]. The peak Mn concentration at 10 cm is likely attributed to its redox-sensitive nature and shifts in bioavailability induced by root activity. In contrast, Pb exhibited a steady decline with distance, indicating its limited mobility and strong adsorption into soil particles near the root zone. The ability of *M. floridulus* to alter soil metal dynamics underscores its potential for phytoremediation in coal gangue dumps. Its deep and extensive root system facilitates metal stabilization through rhizofiltration and rhizosphere modifications, making it an effective candidate for ecological restoration in contaminated soils [31,32].

The findings suggest that plant-assisted remediation with *M. floridulus* plays a crucial role in heavy metal redistribution and stabilization over time, offering valuable insights for ecological risk mitigation. The ability of *M. floridulus* to enhance metal retention near root zones reduces potential leaching risks, while long-term site closure promotes the gradual stabilization of Cr, Ni, and Mn, lowering their ecological mobility [32,33]. Additionally, organic matter accumulation and increased microbial activity in the rhizosphere contribute to heavy metal sequestration, improving overall soil quality. The effectiveness of phytoremediation strengthens over time, emphasizing the significance of plant-based restoration strategies. To optimize remediation efforts, establishing vegetation cover on coal gangue dumps can accelerate metal stabilization, while site-specific monitoring of heavy metal bioavailability remains essential for sustainable ecological management [34].

### 3.2. Evaluation of Soil Heavy Metal Contamination and Associated Ecological Risks at the Three Study Sites

The findings from the single-factor pollution index (Table 1) emphasize the significant role of mine closure duration in shaping the dynamics of heavy metal contamination across the three coal gangue dumps. As observed, prolonged mine closure results in the increased accumulation of certain heavy metals, such as Cu and Pb, while others, such as Cr and Mn, showed signs of natural recovery. This indicates that mine closure alone may not be sufficient to fully mitigate heavy metal risks, and additional remediation measures could be necessary to aid the recovery of mining-impacted soils [35].

The results from the Nemero comprehensive pollution index (Table 2) align with the single-factor pollution index, confirming that Cd remains the dominant pollutant at all study sites. The content of Cd at all plots was classified as severe, with levels far exceeding background values. This trend is consistent with other mining-impacted areas, where Cd tends to accumulate over extended periods [3,36]. The persistence of Cd in soils is largely attributed to its high mobility and bioavailability, which allow it to remain in the environment for prolonged durations, making it a critical pollutant that demands immediate attention [36]. While Cd poses the greatest ecological risk, the other metals—Cr, Mn, Ni, Cu, and Pb—demonstrated varying levels of contamination across the sites. Mn at site C was the only element that did not exceed pollution thresholds, suggesting that either Mn contamination is less severe at this site or natural attenuation processes have been more effective at reducing its concentration [4]. The contamination levels of Cr, Ni, Cu, and Pb mirrored the trends observed in the single-factor index. These consistent patterns highlight the progressive accumulation of Cu and Pb, especially in areas with longer mine closure durations, suggesting a buildup effect over time. Previous studies have demonstrated that Pb and Cu tend to accumulate over time in mining regions due to their relatively low mobility and the ongoing deposition of mining residues [37]. The assessment of soil heavy metal contamination and potential ecological risks highlights site-specific variations in Ni and Cr dynamics, influenced by environmental factors and remediation processes. Ni content exhibited fluctuating trends, with an initial increase at Plot B due to residual contamination from coal waste, followed by a decline at Plot C, likely resulting from plant uptake and sequestration in organic-rich fractions. In contrast, Ni remained stable at Plot A, where continuous gangue deposition limited significant remediation. The mobility of Ni is closely linked to soil pH, microbial activity, and its association with iron and manganese oxides, which can influence its release or immobilization [38]. Similarly, Cr content initially increased before declining, suggesting natural attenuation processes such as leaching, weathering, and adsorption onto soil particles, which gradually reduce its bioavailability over time [39]. These findings underscore the importance of monitoring site-specific soil chemistry to assess heavy metal risks and optimize phytoremediation strategies. The results from both the single-factor and Nemero comprehensive indices underscore the crucial role of mine closure duration in determining the persistence and mobility of these metals in the environment. The progressive accumulation of Cu and Pb is especially concerning due to their significant ecological risks, particularly for soil health and surrounding ecosystems. Studies have shown that Pb is toxic to plants, animals, and humans, and its accumulation in soils can lead to bioaccumulation in food chains [37]. Likewise, while Cu is essential for plant growth, it becomes toxic at elevated levels, exacerbating environmental risks in coal gangue-dominated regions [37]. These findings highlight the need for effective management and remediation strategies to mitigate the ecological hazards associated with these metals in mining-impacted areas.

The results for the potential ecological hazard index (Table 3 and Table 4) highlight Cd as the most significant ecological threat across all study sites, with its risk indices far exceeding those of other heavy metals. The individual ecological risk index (Eri) values for Cd at Plot A (Anyuan Coal Mine) (1936.9), Plot B (Gaokeng Coal Mine) (767.22), and Plot C (Qingshan Coal Mine) (1174) underscore the severity of its impact, consistently identifying Cd as a critical pollutant in mining-affected soils [20]. The cumulative ecological risk indices (RI) further reinforce this pattern, with values of 9684.5 for Plot A, 3836.1 for Plot B, and 5870 for Plot C, clearly establishing Cd as the predominant environmental stressor in the coal gangue dump sites under study. These findings align with prior research, which has consistently identified Cd as a highly toxic and persistent pollutant in mining regions [3]. Cd’s mobility and bioavailability in soils enable it to persist and accumulate over extended periods, posing significant long-term risks to soil health, vegetation, and overall ecosystem stability [40,41]. At Plot A, the elevated Cd levels are likely linked to ongoing mining activities, which contribute to the continuous accumulation of this metal. In contrast, lower levels of Cd at Plots B and C suggest potential natural attenuation processes at work. Nonetheless, the persistence of Cd contamination, even years after mine closure, highlights the challenge of mitigating its environmental impact once deposited [42]. The other metals, Cr, Mn, Ni, Cu, and Pb, exhibited moderate to low ecological risks, with individual risk indices significantly lower than those of Cd. Notably, Ni and Cu showed higher content at Plot C, indicating their more substantial role in contributing to ecological risk at this site, though their contributions remained considerably lower than Cd. While Ni and Cu are essential micronutrients for plants, excessive content can be toxic, and the higher levels of these metals at Plot C likely reflect residual mining impacts. These elements tend to accumulate more in soils with lower mobility compared to Cd [43,44]. Mn and Cr presented minimal ecological risks across all sites, with their lower risk indices suggesting that unlike cadmium (Cd), their environmental impact may be lessened by natural processes. These metals showed less persistence in the environment, likely due to their lower bioavailability and the greater ability of natural attenuation mechanisms to reduce their content over time. Natural attenuation processes such as leaching, sorption, and redox transformations may play a significant role in reducing the mobility and bioavailability of these metals in contaminated soils. Previous studies have indicated that Cr and Mn content in soils impacted by mining can decrease after extended closure periods, suggesting that these metals may be less persistent than Cd and more likely to undergo natural attenuation [4,43,44]. This trend aligns with the general understanding that Cr and Mn, unlike Cd, are less susceptible to long-term accumulation in soils due to their chemical behavior and the relative ease with which they can be immobilized or transformed into less toxic forms. In summary, while Cd remains the dominant ecological threat across all sites, the varying trends in the contamination and ecological risk of other metals, such as Cu and Pb, suggest ongoing challenges in mitigating the impacts of mining residues. The continuous accumulation of Cu and Pb, especially at sites with longer mine closure periods, underscores the need for targeted remediation strategies to address these persistent pollutants and safeguard ecosystem health.

## 4. Materials and Methods

### 4.1. Study Site

**Figure 2:**
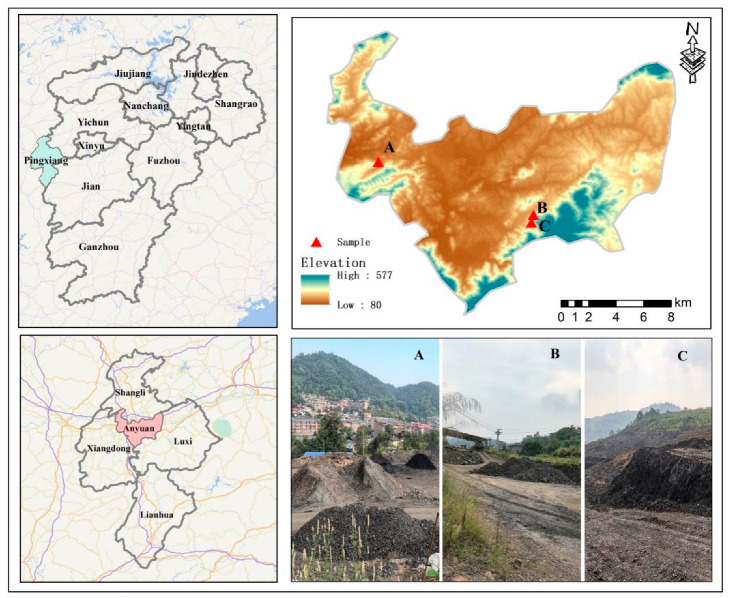
Map of the study area showing the geographic location of the study area in Pingxiang City, Jiangxi Province, and highlighting the sites of the Anyuan, Gaokeng, and Qingshan coal mines. The coordinates of each site are marked, along with surrounding features and the types of natural vegetation. The pictures show the three coal mine sites in Pingxiang City, Jiangxi Province: Anyuan Coal Mine (0-year abandonment, continuously active with coal gangue accumulation) (**A**), Gaokeng Coal Mine (8-year abandonment, mining ceased 8 years ago) (**B**), and Qingshan Coal Mine (12-year abandonment, mining ceased 12 years ago) (**C**).

The study was conducted in Pingxiang City, situated in the western part of Jiangxi Province of China (113°35′–114°17′ E, 27°20′–28°0′ N) (Figure 2). The area experiences a subtropical humid monsoon climate with distinct seasons, abundant sunlight, and significant rainfall. Extreme temperatures occur in January and July, reaching lows of −9.3 °C and highs of 41 °C, respectively, with an average annual temperature of 17.3 °C. Rainfall is concentrated between April and May, contributing to an average annual precipitation of 1603.2 mm. The predominant soil type is sandy, composed of rocks such as carbonaceous shale, carbonaceous sandstone, sandstone, shale, clay, etc. The soil texture is sandy, with a pH range of 7.5–8.5 and soil organic matter ranging from 4 to 36.42 g kg^−1^. This study focused on the Anyuan Coal Mine, Gaokeng Coal Mine, and Qingshan Coal Mine, all under the jurisdiction of the Pingxiang Mining Group. As shown in Table 5 and Figure 2, most areas of the Anyuan Coal Mine coal gangue dump remain bare and operational, exhibiting limited vegetation cover. The Gaokeng Coal Mine coal gangue dump has been out of operation for approximately eight years and remains unremediated, allowing the natural growth of shrubs such as *M. floridulus* and *Xanthium strumarium*. In contrast, the Qingshan Coal Mine, which ceased operations about twelve years ago, has undergone some remediation and now supports the natural growth of various herbaceous plants and shrubs, including *M. floridulus*, *Elodea canadensis*, and *X. strumarium* (Table 5 and Figure 2).

### 4.2. Experimental Design

The study was conducted using a split-plot design. The main factor was the three large state-owned coal mines in Pingxiang City, Jiangxi Province, selected based on their years of abandonment: Anyuan Coal Mine (0 years, still active with ongoing coal gangue accumulation), Gaokeng Coal Mine (8 years, coal mining ceased 8 years ago), and Qingshan Coal Mine (12 years, coal mining ceased 12 years ago). These sites, designated A, B, and C, have experienced decades of coal mining, resulting in large coal gangue dumps that pose potential risks of heavy metal contamination to the surrounding soil. *M. floridulus*, a perennial grass known for its high tolerance to Cd, Zn, and Pb, was chosen as the representative plant due to its resilient growth and dominance at all three sites, despite significant anthropogenic disturbances. Sampling was conducted during the growing season (April 2024), with sub-factors being soil type (rhizosphere vs. non-rhizosphere), distance from the roots (5 cm, 10 cm, 20 cm, and 50 cm), and the analysis of root exudates, to determine heavy metal content. Emphasis was placed on the topsoil (<10 cm) to assess the influence of root activity on soil properties and potential heavy metal contamination. Soil pollution risk was evaluated using the single-factor pollution index (Table 6), Nemero comprehensive pollution index, and potential ecological risk index (Table 7).

### 4.3. Collecting Root Exudates

**Figure 3:**
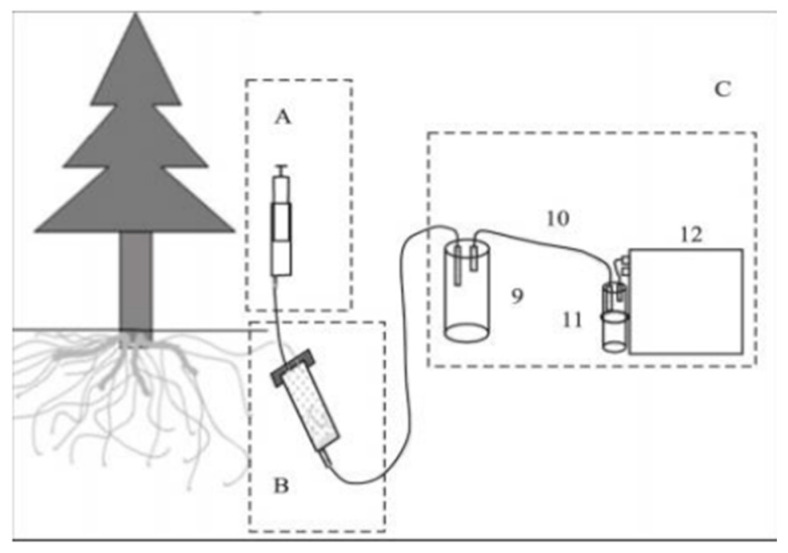
Schematic of the components of the root exudate sampling device. Component A is the inlet unit, B is the collection unit, and C is the filtration unit. The device also includes a filtration bottle (9), a silicone tube (10), a drying tube (11), and a vacuum pump (12).

Root exudates were collected using a static in situ collection device (Figure 3). The collection process began by selecting healthy plants with sparse surrounding vegetation for sampling. Root excavation was performed by carefully digging around the base of the selected plant until a live root was uncovered. The excavation continued along the direction of root extension, targeting roots between 15 cm and 20 cm in length [47]. In cases where longer roots were present, lateral roots of similar length were selected. Throughout the process, care was taken to minimize damage to the root system to ensure the roots remained viable. The excavation area was limited to less than one-fourth of the circular area surrounding the plant to further protect the root structure.

After excavation, the root washing procedure involved rinsing the extracted roots in situ with distilled water to remove any adhering debris. Once cleaned, the roots were placed into a syringe (device B) lined with glass wool at the bottom for support. Sterile glass beads, approximately 1 mm in diameter, were added as a cultivation substrate. To prevent contamination, the syringe was wrapped with sealing film and covered with aluminum foil. A second syringe (device A) was then filled with distilled water and injected into device B. This setup was incubated for 20–30 min [47]. Finally, a vacuum pump was used to filter and collect the root exudates into device C.

### 4.4. Measurement Methods

#### 4.4.1. Qualitative Determination of Root Exudates

The procedure for determining root exudates was adapted from Wang et al. [42]. A 10 mL sample of root exudate extract was filtered through a 0.45 μm membrane and mixed with an equal volume of chromatographic-grade CH_2_Cl_2_. The mixture was agitated for 2 h, followed by phase separation to isolate the lower layer. This layer was concentrated using a rotary evaporator under reduced pressure until the volume reached approximately 1 mL. The final volume was then adjusted to 1.5 mL with additional chromatographic-grade CH_2_Cl_2_, and the sample was prepared for analysis using gas chromatography–mass spectrometry (GC-MS). GC-MS analysis was conducted using a DB-5ms capillary column (30 m × 0.25 mm, film thickness: 0.25 μm). The injection port temperature was set to 280 °C, while the initial column temperature was 50 °C, held for 3 min. The temperature then increased to 290 °C at a rate of 10 °C/min, held for 20 min. Helium served as the carrier gas, flowing at 1 mL/min with an injection volume of 1 μL. The mass spectrometer employed electron impact ionization at 70 eV, scanning within the mass-to-charge (*m*/*z*) range of 35–800 AMU at a speed of 0.2 s per scan. The ion source temperature was set at 200 °C, with the interface temperature at 250 °C and a solvent cut time of 3 min. Compound identification was achieved by comparing the total ion chromatograms to standard spectra from the NIST 107 mass spectral database using computer-assisted analysis.

#### 4.4.2. Determination of Heavy Metal Content in Soil

The content of Mn, Cd, Cr, Ni, Cu, and Pb in the soil was determined using graphite furnace digestion followed by inductively coupled plasma mass spectrometry (ICP-MS) [48]. Soil samples were air-dried, ground, and sieved through a 100-mesh filter. A 0.3 g portion of the sample (accurate to 0.001 g) was placed in a digestion vessel, to which 6 mL of nitric acid, 3 mL of hydrochloric acid, and 2 mL of hydrofluoric acid were added in sequence, followed by gentle mixing. The samples were then subjected to graphite furnace digestion at 180 °C for 100 min. After digestion, the resulting solution was filtered into a 25 mL volumetric flask, brought to volume, and thoroughly mixed. The mixture was allowed to settle for 60 min, after which the supernatant was filtered through a 0.45 μm membrane and analyzed using ICP-MS.

#### 4.4.3. Soil Heavy Metal Pollution Assessment Methods

The assessment of soil heavy metal pollution in the study area was conducted using the single-factor index method [45], the Nemero comprehensive pollution index method [46], and the potential ecological hazard index method [49].

The single-factor pollution index method evaluates soil quality by analyzing the ratio of measured heavy metal content in the soil to reference values. The classification standards are shown in Table 6, and the calculation results are presented in Table 1. The formula used for this assessment is:

(1) $$P_{i}=\frac{C_{i}}{S_{i}}$$

where P_i_ represents the single pollution index of pollutant ii in the soil; C_i_ is the measured concentration of pollutant ii in the soil; and S_i_ is the evaluation standard for pollutant i.

The reference values used for this evaluation were based on the soil environmental background values of Jiangxi Province, where the content of Mn, Cd, Cr, Ni, Cu, and Pb is 259.00 mgkg^−1^, 0.10 mg kg^−1^, 48.00 mg kg^−1^, 19.00 mg kg^−1^, 20.80 mg kg^−1^, and 32.10 mg kg^−1^, respectively.

The Nemero comprehensive pollution index is defined as the average of the heavy metal content in the soil at the study site, calculated alongside the maximum single pollution index. This index provides a comprehensive reflection of the degree of soil pollution. The classification standards are shown in Table 7, and the calculation results are presented in Table 2. The formula is as follows:

(2) $$P_{N}=\sqrt{\frac{P_{ave}^{2}+P_{max}^{2}}{2}}$$

where P_N_ is the comprehensive pollution index; P_ave_ is the average value of the individual pollution indices at the sampling points; and P_max_ is the maximum value of the individual pollution indices at the sampling points.

The potential ecological hazard index (PEHI) method is used to evaluate heavy metal pollution in soil and sediments. It reflects both the pollution level of individual heavy metal contaminants and the combined effects of multiple heavy metal pollutants. The classification standards are shown in Table 8, and the calculation results are presented in Table 3 and Table 4. The calculation formula is as follows:

(3) $$\mathrm{RI}=\sum E_{r}^{i};\;E_{r}^{i}=T_{r}^{i}\times C_{f}^{i};\;C_{f}^{i}=(C^{i}\;{\mathrm{Ci}}_{\mathrm{measured}})/C_{n}^{i}$$

where RI represents the comprehensive potential ecological risk index for multiple heavy metals in the soil; E_ri_ is the potential ecological hazard coefficient for heavy metal element i, while T_ri_ is the toxicity response factor for element i; and C_if_ denotes the pollution parameter for heavy metal element i. The term C_imeasured_ refers to the measured concentration of heavy metal element i, and C_in_ is the evaluation standard for element i.

### 4.5. Data Analysis

Data collected from the three coal mine sites in Pingxiang City, Jiangxi Province—Anyuan Coal Mine (0-year abandonment), Gaokeng Coal Mine (8-year abandonment), and Qingshan Coal Mine (12-year abandonment)—were analyzed to assess the impact of abandonment duration on root exudates and soil properties. Three healthy individuals of the dominant plant species *M. floridulus* were selected for sampling at each site. Root exudates and soil samples were collected from the rhizosphere (0 cm depth) and non-rhizosphere soil at depths of 5 cm, 10 cm, 20 cm, and 50 cm, with a focus on the topsoil (<10 cm). Sampling took place from 9 April to 26 April 2024 to account for seasonal effects. Analysis of variance (ANOVA) was conducted to assess differences among the sites and soil depths. Data were log-transformed to meet the assumptions of normality and homoscedasticity. Significant results from the ANOVA were followed by Tukey’s honest significant difference (HSD) test for post hoc analysis. Graphical representations were created using Origin software (Origin 2023 (10.0)), and data analysis was performed using SPSS software (SPSS Version 16.0).

## 5. Conclusions

This study assessed heavy metal contamination in soils from three large coal mines with closure durations of 0, 8, and 12 years in relation to root exudates from *M. floridulus*. The results showed significant variations in contamination across the sites, with Cd being the most critical pollutant, particularly at Anyuan Coal Mine. The Nemero comprehensive pollution index and potential ecological hazard index confirmed that Cd posed the greatest ecological risk. Cu and Pb levels increased with mine closure duration, indicating accumulation, while Cr and Mn levels showed natural attenuation over time. The study found that *M. floridulus* influenced the distribution of heavy metals in the soil, with higher content near the roots, suggesting that rhizosphere processes can modulate metal mobility and bioavailability. This points to the potential of using plant-mediated processes for phytoremediation. The study’s novel contribution lies in examining how mine closure duration and rhizosphere dynamics interact to affect heavy metal contamination in coal gangue dump soils. It emphasizes the need for site-specific, long-term remediation strategies. The findings suggest that *M. floridulus* can be used as a bioindicator and bioremediator in coal mine environments. In summary, targeted remediation, especially for Cd, is essential, and ongoing monitoring of post-mining landscapes is necessary. Future research should focus on the role of root exudates in enhancing metal mobilization and exploring phytoremediation techniques in coal gangue dumps for sustainable mine reclamation.

## Figures and Tables

**Table 1:** Single-factor pollution index analysis *.

Research Area	Cr	Mn	Ni	Cu	Cd	Pb
A	1.238 a	1.736 a	2.276 b	1.472 b	64.56 a	2.286 b
B	5.17 b	1.934 a	1.794 a	2.302 b	25.574 c	2.306 b
C	1.166 a	0.648 b	2.84 c	4.32 a	39.134 b	11.444 a

* A, B, and C represent the Anyuan Coal Mine, Gaokeng Coal Mine, and Qingshan Coal Mine coal gangue dumps in Pingxiang City, respectively. Different lowercase letters (a, b, and c) indicate significant differences among the A, B, and C sites (*p* < 0.05), while the same letter indicates no significant differences among the A, B, and C sites (*p* > 0.05).

**Table 2:** Nemero comprehensive pollution index analysis *.

Research Area	Cr	Mn	Ni	Cu	Cd	Pb
A	1.41 a	1.78 a	2.54 b	1.52 b	152.00 a	2.81 b
B	2.27 b	2.16 a	1.99 a	2.45 b	31.57 c	2.85 b
C	1.37 a	0.73 b	3.47 c	5.81 a	41.74 b	16.64 a

* A, B, and C represent the Anyuan Coal Mine, Gaokeng Coal Mine, and Qingshan Coal Mine coal gangue dumps in Pingxiang City, respectively. Different lowercase letters (a, b, and c) indicate significant differences among the A, B, and C sites (*p* < 0.05), while the same letter indicates no significant differences among the A, B, and C sites (*p* > 0.05).

**Table 3:** Potential ecological hazard index evaluation results *.

Research Area	Cr	Mn	Ni	Cu	Cd	Pb
A	2.48 a	1.74 a	11.38 b	7.36 b	1936.9 a	11.43 b
B	4.14 b	1.93 a	8.98 a	11.52 b	767.22 c	11.53 b
C	2.33 a	0.65 ab	14.19 c	21.61 a	1174 b	57.2 a

* A, B, and C represent the Anyuan Coal Mine, Gaokeng Coal Mine, and Qingshan Coal Mine coal gangue dumps in Pingxiang City, respectively. Different lowercase letters (a, b, and c) indicate significant differences among the A, B, and C sites (*p* < 0.05), while the same letter indicates no significant differences among the A, B, and C sites (*p* > 0.05).

**Table 4:** Comprehensive potential ecological hazard index evaluation results *.

Study Area	Cr	Mn	Ni	Cu	Cd	Pb
A	12.4 a	8.7 a	56.9 b	36.8 b	9684.5 a	57.15 b
B	20.7 b	9.65 a	44.9 a	57.6 b	3836.1 c	57.65 b
C	11.65 a	3.25 b	70.95 c	108.05 a	5870 b	286 a

* A, B, and C represent the Anyuan Coal Mine, Gaokeng Coal Mine, and Qingshan Coal Mine coal gangue dumps in Pingxiang City, respectively. Different lowercase letters (a, b, and c) indicate significant differences among the A, B, and C sites (*p* < 0.05), while the same letter indicates no significant differences among the A, B, and C sites (*p* > 0.05).

**Table 5:** Geographic information of the study area.

Site	Closure Time	Location	Geographic Coordinates	Dominant Vegetation
A	0 years	Anyuan Coal Mine gangue dump	27°36′22″ N, 113°53′55″ E	Sparse *M. floridulus*
B	8 years	Gaokeng Coal Mine gangue dump	27°36′4″ N, 113°53′50″ E	*M. floridulus*, *X. strumarium*
C	12 years	Qingshan Coal Mine gangue dump	27°38′27″ N, 113°47′49″ E	*M. floridulus*, *E. canadensis*

**Table 6:** Soil single-level pollution grading standards *.

*p*	*p* ≤ 1	1 < *p* ≤ 2	2 < *p* ≤ 3	*p* > 3
Pollution Level	Non-Polluted	Light Pollution	Moderate Pollution	Heavy Pollution

* From [45].

**Table 7:** Classification standards for soil comprehensive pollution levels *.

Soil Comprehensive Pollution Level	Soil Comprehensive Pollution Index	Pollution Degree	Pollution Status
1	*p* ≤ 0.7	Safe	Clean
2	0.7 < *p* ≤ 1.0	Warning Line	Relatively clean
3	1.0 < *p* ≤ 2.0	Light Pollution	Pollutants exceed initial pollution value, crops begin to be affected
4	2.0 < *p* ≤ 3.0	Moderate Pollution	Significant soil and crop pollution
5	*p* > 3.0	Heavy Pollution	Severe soil and crop pollution

* From [46].

**Table 8:** Evaluation of comprehensive potential ecological risk index *.

E_ri_	Risk Level	RI	Hazard Grade
<40	Minor	<150	Minor
40 ≤ E_ri_ < 80	Moderate	150 ≤ RI < 300	Moderate
80 ≤ E_ri_ < 160	High	300 ≤ RI < 600	High
160 ≤ E_ri_ < 320	Very High	≥600	Very High
≥320	Extremely High		

* From [49].

## Data Availability

The data presented in this study are available upon request from the corresponding author.

## References

[1] Shang Y., Sang N. (2022). Pollution Characteristics and Phytotoxicity of Heavy Metals in the Soil Around Coal Gangue Accumulation Area. Environ. Sci..

[2] Wuana R., Okieimen F. (2011). Heavy Metals in Contaminated Soils: A Review of Sources, Chemistry, Risks and Best Available Strategies for Remediation. ISRN Ecol..

[3] Zhang Y., Cao Y., Feng N., Liu Y., Zhang Y., Wang Q., Liu J. (2024). Risk assessment of heavy metals in the soil of an abandoned coal mine area. J. China Coal Soc..

[4] Cao J., Liu Y., Guo G. (2004). The current situation in comprehensive utilization of gangue. Chin. J. Environ. Eng..

[5] Jia M. (2019). The Current Situation Research on Comprehensive Utilization of Coal Gangue. Conserv. Util. Miner. Resour..

[6] Gao S., Zhao T. (2022). Heavy Metal Stress in Coal Gangue Dumps and Plant Adaptations. Sci. Total Environ..

[7] Wang Y. (2022). Status and prospect of harmless disposal and resource comprehensive utilization of solid waste of coal gangue. Coal Geol. Explor..

[8] Ghosh M., Singh S. (2005). A review on phytoremediation of heavy metals and utilization of its byproducts. Appl. Ecol. Environ. Res..

[9] Shah V., Dani P., Daverey A. (2024). Phytoremediation of heavy metal contaminated soil using Bidens pilosa: Effect of varying concentrations of sophorolipids. Appl. Biochem. Biotechnol..

[10] Ali H., Khan E., Sajad M. (2013). Phytoremediation of heavy metals—Concepts and applications. Chemosphere.

[11] Zheng X. (2004). System Structure and Fuzzy Evaluation of Sustainable Development in Mining Area. Met. Mine.

[12] Raskin I., Smith R., Salt D. (1994). Phytoremediation of metals: Using plants to remove pollutants from the environment. Environ. Sci. Technol..

[13] Liu Y., Chen Y., Li Y., Ding C., Li B., Han H., Chen Z. (2024). Plant growth-promoting bacteria improve the Cd phytoremediation efficiency of soils contaminated with PE–Cd complex pollution by influencing the rhizosphere microbiome of sorghum. J. Hazard. Mater..

[14] Miller M., Sweeney R. (2016). Combining phytoremediation and bioremediation: A promising approach to soil decontamination. Environ. Manag..

[15] Mudgal V., Raninga M., Patel D., Ankoliya D., Mudgal A. (2023). A review on Phytoremediation: Sustainable method for removal of heavy metals. Mater. Today Proc..

[16] Guan C., Fu W., Zhang X., Li Z., Zhu Y., Chen F., Ji J., Wang G., Gao X. (2023). Enhanced phytoremediation efficiency of PHE-contaminated soil by rape (*Brassica napus* L.) assisted with PHE-degradable PGPR through modulating rhizobacterial communities. Ind. Crops Prod..

[17] Zhao F., Wang Y., Zhang J., Wang S. (2020). Assessing the impact of phytoremediation on heavy metal accumulation in Miscanthus floridulus. Environ. Monit. Assess.

[18] Chen J., Chen Z., Zhang W. (2021). The role of Miscanthus floridulus in the phytoremediation of heavy metal-contaminated soils. Environ. Sci. Pollut. Res..

[19] Salt D., Smith R., Raskin I. (1998). Phytoremediation. Annu. Rev. Plant Biol..

[20] Vangronsveld J., Herzig R., Weyens N., Boulet J., Adriaensen K., Ruttens A., Mench M. (2009). Phytoremediation of contaminated soils and groundwater: Lessons from the field. Environ. Sci. Pollut. Res..

[21] Prasad S., Yadav K., Kumar S., Gupta N., Cabral-Pinto M., Rezania S., Radwan N., Alam J. (2021). Chromium contamination and effect on environmental health and its remediation: A sustainable approache. J. Environ. Manag..

[22] Rouhani A., Skousen J., Tack F. (2023). An overview of soil pollution and remediation strategies in coal mining regions. Minerals.

[23] D’Orazio M., Campanella B., Bramanti E., Ghezzi L., Onor M., Vianello G., Vittori-Antisari L., Petrini R. (2020). Thallium pollution in water, soils and plants from a past-mining site of Tuscany: Sources, transfer processes and toxicity. J. Geochem. Explor..

[24] Xiao Y., Chen L., Teng K., Ma J., Xiang S., Jiang L., Liu G., Yang B., Fang J. (2023). Potential roles of the rhizospheric bacterial community in assisting Miscanthus floridulus in remediating multi-metal (loid) s contaminated soils. Environ. Res..

[25] Mocek-Płóciniak A., Mencel J., Zakrzewski W., Roszkowski S. (2023). Phytoremediation as an effective remedy for removing trace elements from ecosystems. Plants.

[26] Emurotu J., Azike E., Emurotu O., Umar Y. (2024). Chemical fractionation and mobility of Cd, Mn, Ni, and Pb in farmland soils near a ceramics company. Environ. Geochem. Health.

[27] Montiel-Rozas M., Madejón E., Madejón P. (2016). Effect of heavy metals and organic matter on root exudates (low molecular weight organic acids) of herbaceous species: An assessment in sand and soil conditions under different levels of contamination. Environ. Pollut..

[28] Noreen S., Malik Z., Luqman M., Fatima I., Tahir U.A., Dar M., Rizwan M. (2024). Effect of bacillus strain and Fe-modified biochar on lead (Pb) bioaccumulation and oxidative stress in wheat (*Triticum aestivum* L.) grown in Pb contaminated soil. S. Afr. J. Bot..

[29] Bali A., Sidhu G., Kumar V. (2020). Root exudates ameliorate cadmium tolerance in plants: A review. Environ. Chem. Lett..

[30] Mishra J., Singh R., Arora N. (2017). Alleviation of heavy metal stress in plants and remediation of soil by rhizosphere microorganisms. Front. Microbiol..

[31] Pidlisnyuk V., Stefanovska T., Lewis E., Erickson L., Davis L. (2014). Miscanthus as a productive biofuel crop for phytoremediation. Crit. Rev. Plant Sci..

[32] Wu B., Luo S., Luo H., Huang H., Xu F., Feng S., Xu H. (2022). Improved phytoremediation of heavy metal contaminated soils by Miscanthus floridulus under a varied rhizosphere ecological characteristic. Sci. Total Environ..

[33] Padhye L., Srivastava P., Jasemizad T., Bolan S., Hou D., Shaheen S., Rinklebe J., O’Connor D., Lamb D., Wang H. (2023). Contaminant containment for sustainable remediation of persistent contaminants in soil and groundwater. J. Hazard. Mater..

[34] Bandyopadhyay S. (2022). Plant-assisted metal remediation in mine-degraded land: A scientometric review. Int. J. Environ. Sci. Technol..

[35] Ren H., Wang J., Cao H., Zhou W., Zhang X. (2006). Phytoavailable lead in rhizosphere of lettuce. Environ. Sci..

[36] Li M., Herong G. (2017). Characteristics and Source Identification of Heavy Metals in Subsidence Lake in Zhuxianzhuang Coal Mine in the North of Anhui Province, China. Earth Environ..

[37] Zheng Y., Zang Z., Yao D., Chen X. (2013). Characteristics of temporal-spatial distribution and enrichment of heavy metals in coal mine reclaimed soil. J. China Coal Soc..

[38] Palansooriya K., Shaheen S., Chen S., Tsang D., Hashimoto Y., Hou D., Bolan N., Rinklebe J., Ok Y. (2020). Soil amendments for immobilization of potentially toxic elements in contaminated soils: A critical review. Environ. Int..

[39] Dhal B., Thatoi H., Das N., Pandey B. (2013). Chemical and microbial remediation of hexavalent chromium from contaminated soil and mining/metallurgical solid waste: A review. J. Hazard. Mater..

[40] Mohan A., Raiput A., Singh A., Steele P., Pittman C. (2011). Modeling and evaluation of chromium remediation from water using low cost biochar, a green adsorbent. J. Hazard. Mater..

[41] Edelstein M., Ben-Hur M. (2018). Heavy metals and metalloids: Sources, risks and strategies to reduce their accumulation in horticultural crops. Sci. Hortic..

[42] Wang J., Farooq T., Aslam A., Shakoor A., Chen X., Yan W. (2021). Non-targeted metabolomics reveal the impact of phenanthrene stress on root exudates of ten urban greening tree species. Environ. Res..

[43] Li W., Ren X., Cai T. (2011). Assessment of Nutrient Content and Heavy Metal Pollution in Gangue Waste Lands with Different Dumping Years. Sci. Silvae Sin..

[44] Liu F., Chen Z., Liu Y., Zhu J., Bo T. (2020). Effects of natural vegetation restoration on Fe/Mn leaching and migration in coal gangue yard. Bull. Soil Water Conserv..

[45] Zheng H., Chen J., Deng W., Tan m. (2005). Assessment of soil heavy metals pollution in the chemical industrial areas of Nanjing peri-urban zone. Acta Sci. Circumstantiae.

[46] Brady J., Ayoko G., Martens W., Goonetilleke A. (2015). Development of a hybrid pollution index for heavy metals in marine and estuarine sediments. Environ. Monit. Assess..

[47] Guo W., Zhang Z., Liu Q., Yin H. (2019). Research progress of root exudates collection technology. Chin. J. Appl. Ecol..

[48] Long J., Tan J., Wu Y., Zhu Y., Xu X. (2013). A Comparative Study on the Detection of Heavy Metal in Soil with Different Digestion Methods. Environ. Monit. China.

[49] Xu Z., Ni S., Tuo X., Zhang C. (2008). Calculation of Heavy Metals’ Toxicity Coefficient in the Evaluation of Potential Ecological Risk Index. Environ. Sci. Technol..

